# Weight-loss dynamics with tirzepatide versus semaglutide

**DOI:** 10.1093/pnasnexus/pgag171

**Published:** 2026-06-16

**Authors:** A J Venkatakrishnan, Karthik Murugadoss, Venky Soundararajan

**Affiliations:** Metabolism Agentic Intelligence Atlas (MAIA), nference, 1 Main Street, East Arcade Building, Cambridge, MA 02142, USA; Metabolism Agentic Intelligence Atlas (MAIA), nference, 1 Main Street, East Arcade Building, Cambridge, MA 02142, USA; Metabolism Agentic Intelligence Atlas (MAIA), nference, 1 Main Street, East Arcade Building, Cambridge, MA 02142, USA

**Keywords:** semaglutide, tirzepatide, real-world evidence, weight loss, effectiveness

## Abstract

Glucagon-like peptide-1 receptor agonists (GLP-1RAs) are widely used for obesity and type 2 diabetes, yet substantial variability in weight-loss response remains poorly understood. We conducted a retrospective cohort study using de-identified electronic health records and performed 1:1 propensity matching on age, sex, type 2 diabetes status, baseline BMI and weight, index year, and follow-up duration. The matched cohorts included 10,339 tirzepatide-treated and 10,339 semaglutide-treated patients. Patients were categorized by maximum weight loss over 2 years into five response groups. Adverse events were identified through AI-enabled curation of clinical notes. Weight-loss trajectories, demographic patterns, adverse-event profiles, and pre- to posttreatment disease-prevalence changes were compared across drugs. Patients treated with tirzepatide lost more weight than those treated with semaglutide (mean reduction, 14.7 vs. 10.8%; *P* < 0.001). High-response rates (≥15% weight loss in year 1) were nearly doubled with tirzepatide (42.6 vs. 21.6%; *P* < 0.001), accompanied by faster monthly weight-loss velocity (2.54 vs. 2.18%). AI-enabled curation showed that tirzepatide was associated with lower prevalence of gastrointestinal and systemic adverse events. For both tirzepatide and semaglutide, women were more represented among high responders than the minimal weight-loss group (<5% weight loss) and White patients were more represented among high responders, whereas Black and Hispanic patients were more represented among the minimal weight-loss group. In this large, propensity-matched real-world cohort, tirzepatide was associated with greater and faster weight loss than semaglutide, with marked demographic variations in outcomes, highlighting the need for next-generation obesity clinical trials and routine care decisions to incorporate the growing body of evidence from widespread use of incretin therapies across diverse patient populations.

Significance statement Glucagon-like peptide-1 receptor agonists have transformed obesity treatment, yet patient responses vary widely and remain insufficiently characterized in real-world settings. In this analysis of large propensity-matched cohorts of patients who were prescribed tirzepatide versus semaglutide, tirzepatide was associated with greater and faster weight loss than semaglutide along with lower clinically documented rates of multiple gastrointestinal and systemic adverse events. Female and White patients on tirzepatide or semaglutide more frequently achieved significant weight loss, while male, Black, and Hispanic patients were overrepresented among patients with minimal weight loss. These findings motivate precision medicine approaches to obesity pharmacotherapy that incorporate real-world treatment and response data.

## Introduction

Incretin-based therapies have transformed obesity treatment by producing clinically meaningful weight loss, yet real-world responses span a wide range (1). Clinical trials and observational cohorts often emphasize average results, but the magnitude of interindividual variability suggests distinct biological subgroups that remain insufficiently defined (2, 3). The characteristics of exceptional responders, the clinical profiles of nonresponders, the pattern of adverse events, and the real-world duration of therapy are not fully understood (3, 4). Electronic health records permit large-scale, longitudinal characterization of weight trajectories and comorbid conditions, although skepticism regarding data quality necessitates careful attention to confounding, population heterogeneity, and ascertainment bias (5–7). AI applied to unstructured notes can augment structured data and improve capture of clinical events (5, 8, 9). In this study, by leveraging propensity-score matching and AI-based curation of clinical documentation, we characterize response heterogeneity, identify physiologic signatures across response groups, and examine differential patterns of adverse events and disease burden changes across tirzepatide and semaglutide.

## Methods

### Data source

This study analyzed de-identified Electronic Health Record (EHR) data from academic medical centers in the United States via the nference nSights Analytics Platform. Prior to analysis, all data underwent expert determination de-identification satisfying Health Insurance Portability and Accountability Act (HIPAA) Privacy Rule requirements [45 Code of Federal Regulations (CFR) §164.514(b)(1)], employing a multilayered transformation approach for both structured data (cryptographic hashing of identifiers, date shifting, and geographic truncation) and unstructured clinical text (ensemble deep learning and rule-based methods with >99% recall for personally identifiable information detection) (10, 11). nference established secure data environments within each participating center, housing these de-identified patient data governed by expert determination. These de-identified data environments were specifically designed to enable data access and analysis without requiring Institutional Review Board (IRB) oversight, approval, or exemption confirmation. Accordingly, informed consent and IRB review were not required for this study.

### Cohort selection

From a longitudinal database of 23 million patients, we identified 640,794 with at least one glucagon-like peptide-1 receptor agonist (GLP-1RA) order. Requiring ≥3 orders spanning ≥1 month yielded 278,471 patients; restricting to semaglutide or tirzepatide prescriptions yielded 215,004 patients. We constructed drug-exclusive cohorts comprising patients who received only semaglutide brands (Ozempic, Wegovy, and Rybelsus) or only tirzepatide brands (Mounjaro and Zepbound); patients could switch between brands of the same drug but not between drugs. We excluded patients with bariatric surgery history and required weight measurements during baseline (≤90 days pretreatment), the first year posttreatment (year 1), and the second year posttreatment (year 2), yielding 34,143 semaglutide-treated and 16,034 tirzepatide-treated patients (Fig. S1).

### Weight trajectories

**Figure 1:**
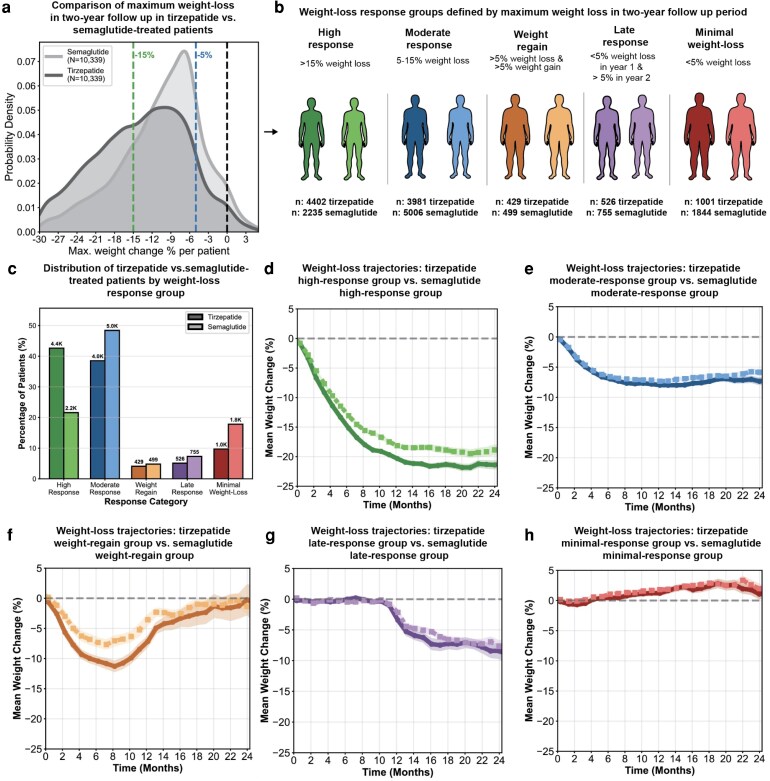
Comparative weight-loss response profiles and longitudinal trajectories in propensity-matched tirzepatide- and semaglutide-treated patients. a) Distribution of maximum weight loss over a 2-year follow-up among tirzepatide- and semaglutide-treated patients after 1:1 propensity matching (*n* = 10,339 per cohort), illustrating a right-shifted distribution for tirzepatide consistent with greater overall weight reduction. b) Five weight-loss response categories based on maximum percentage weight change: high response (>15% loss), moderate response (5–15% loss), weight-regain group (>5% loss in year 1 followed by >5% regain in year 2), late response (<5% loss in year 1 but >5% cumulative loss in year 2), and minimal weight loss (<5% loss). The corresponding counts of tirzepatide- and semaglutide-treated patients in each group are shown. c) Distribution of patients across response categories, demonstrating a higher proportion of high responders with tirzepatide and a higher proportion of minimal responders with semaglutide. d–h) Mean longitudinal weight-change trajectories over 24 months for tirzepatide versus semaglutide within each response category (high, moderate, weight regain, late, and minimal response). Across categories, tirzepatide consistently produces steeper early weight loss and greater sustained reduction, whereas semaglutide shows attenuated initial decline and earlier plateauing. Error bands denote 95% CIs.

Patients in both semaglutide and tirzepatide cohorts were stratified into five response groups (Fig. 1): high response (>15% weight loss during the entire study period), moderate response (5–15% during the entire study period), late response (<5% during year 1, >5% during year 2), weight regain (>5% during year 1, ≥5% regain during year 2), and minimal weight loss (<5% weight loss during the entire study period). The index date was the date of the first GLP-1RA prescription baseline weight was the measurement closest to the index date within 90 days pretreatment. Weight changes were calculated relative to baseline and aggregated into 30-day windows (±15 days). Population-level trajectories were generated by computing patient-level mean percentage changes within each window, then smoothed using Savitzky–Golay filtering (window = 5, polynomial order = 3). Uncertainty was quantified as SEM (SEM = *σ*/√*n*); shaded regions represent ±1 SEM.

### Propensity score matching

To compare treatment responses between tirzepatide and semaglutide while controlling for baseline differences, we performed propensity-score matching for the overall tirzepatide and semaglutide populations as well as separately within each of the five weight-loss outcome categories (high response, moderate response, weight regain, late response, and minimal weight-loss groups). Propensity scores were estimated using multivariable logistic regression with treatment assignment (tirzepatide vs. semaglutide) as the dependent variable and the following covariates: age at index date, sex, type 2 diabetes mellitus (T2DM) status (defined as ≥3 diagnoses within 5 years prior to index), baseline body mass index (BMI; most recent measurement within 90 days before index), baseline weight (most recent measurement within 90 days before index), year of treatment initiation, and follow-up duration in days. Because race itself is not known to be associated with GLP-1RA treatment selection, it was not included as a matching covariate. Nearest-neighbor matching without replacement was performed using a 1:1 matching ratio and a caliper width of 0.1 SDs of the logit of the propensity score. Patients with index dates before January 1, 2023 were excluded to ensure comparable follow-up periods and account for differential market availability of the medications. Balance diagnostics were assessed by comparing standardized mean differences for all matching covariates between matched cohorts within each response category, with values <0.1 considered indicative of adequate balance. We characterized brand name distributions within matched cohorts (Table S1) among the tirzepatide (Mounjaro and Zepbound) and semaglutide (Ozempic, Wegovy, and Rybelsus) branded formulations.

### Statistical analysis

Summary statistics were generated for semaglutide and tirzepatide cohorts across all response groups (Tables S2–S7). Demographics included age at first prescription, gender, and race/ethnicity. Race and ethnicity data were derived from patient self-report documented in the electronic health record. We categorized patients as White (non-Hispanic), Black/African American (non-Hispanic), Hispanic (any race), or other/unknown, with the latter category combining all other racial groups and missing data due to small sample sizes that precluded separate statistical analysis (12). Baseline clinical characteristics included T2DM, BMI, and weight. BMI and weight were defined as the nearest recorded values within 90 days prior to the index date. T2DM status was defined by at least three diabetes diagnostic codes recorded in the 5 years prior to the index date. We characterized weight measurement distributions across a 10-year pretreatment period and the first 2 years posttreatment. GLP-1RA prescription intervals were calculated as the time between the first and last prescription for each patient (Fig. S2), and prescription frequency was defined as the total number of prescriptions received per patient. Follow-up duration was computed as days from index date to last clinical encounter for that patient. Baseline and posttreatment monthly clinical note counts were quantified. Statistical comparisons between propensity-matched tirzepatide and semaglutide cohorts were performed using two-proportion *z*-tests for binary outcomes and χ^2^ tests or Fisher's exact tests (for cell counts <5) for categorical outcomes.

### AI-enabled augmented curation

We employed a multistage information extraction (IE) pipeline using fine-tuned Bidirectional Encoder Representations from Transformers (13) models to extract clinical phenotypes from unstructured clinical notes. The pipeline first applies Named Entity Recognition to identify clinical entities, then uses sequential qualifier models to assess clinical context by determining subject (patient vs. other), temporality (current, past, or hypothetical), and certainty (confirmed, negated, or suspected). This contextualization framework disambiguates confirmed current patient symptoms from negated findings, suspected conditions, or prior diagnoses. This IE approach has been validated in prior studies (8) with F1 scores of 0.93 and 0.95 for conditions and medications, respectively.

### Posttreatment adverse event prevalence analysis

**Figure 2:**
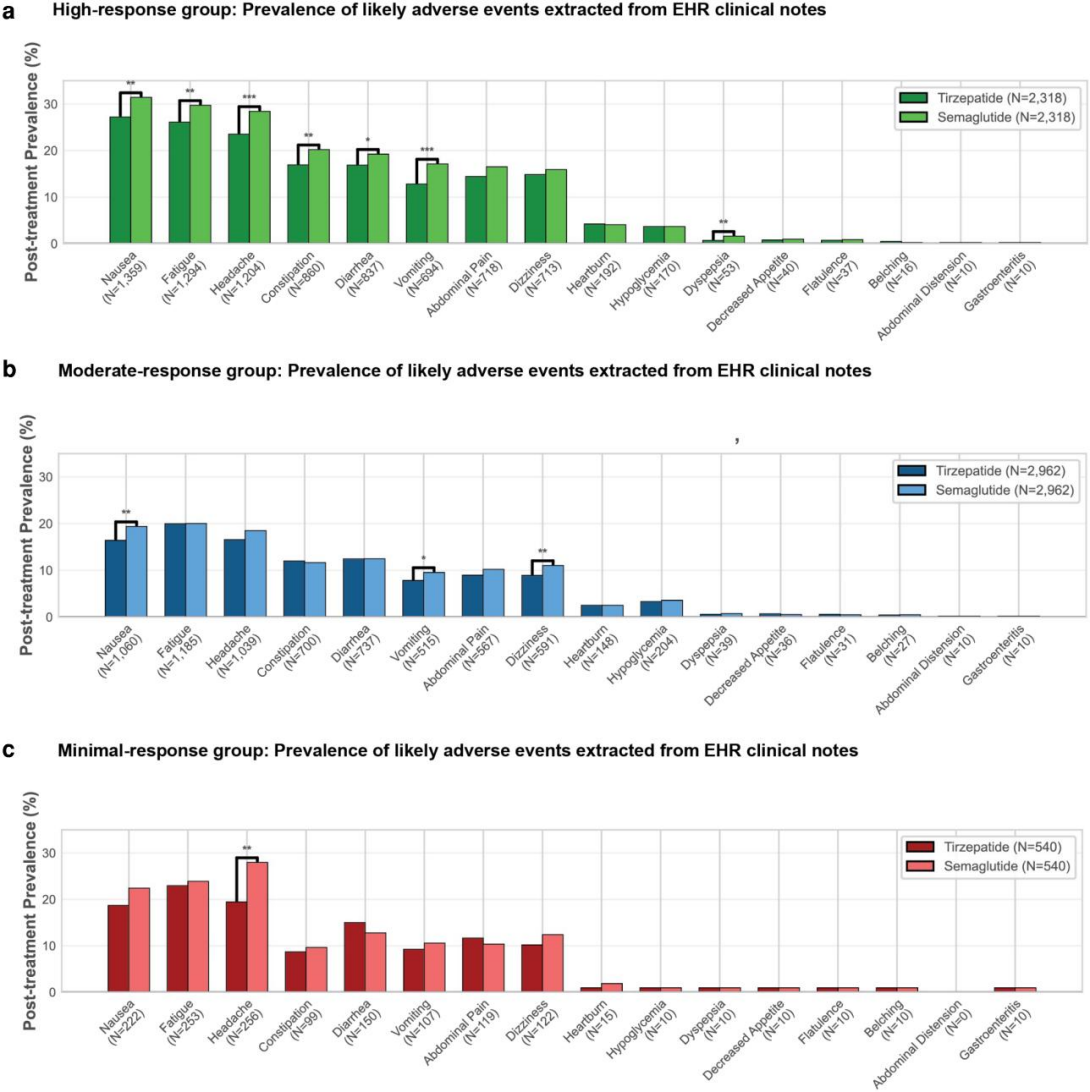
Prevalence of adverse events extracted from unstructured clinical text in tirzepatide- and semaglutide-treated patients across weight-loss response groups. a) Posttreatment prevalence of selected adverse events among patients in the high-response group (*n* = 2,318 per treatment cohort), as identified from unstructured clinical notes. b) Corresponding adverse-event prevalence estimates for the moderate-response group (*n* = 2,962 per cohort). c) Prevalence estimates for the minimal-weight-loss group (*n* = 540 per cohort). For each adverse event, bars represent the proportion of patients with at least one posttreatment mention; asterisks indicate the significance of between-treatment differences.

We performed a comparative analysis of adverse-event prevalence between propensity-matched tirzepatide and semaglutide cohorts within each weight-loss response category. We analyzed 16 commonly reported adverse events identified from Food and Drug Administration prescribing information for both medications, including gastrointestinal, systemic, and metabolic events. Adverse-event prevalence was determined exclusively from unstructured clinical notes during the 2-year posttreatment period using the validated IE pipeline described above. The pipeline identified adverse-event mentions from all available clinical documentation, restricting output to confirmed, current patient-attributed events while excluding negated, suspected, or historical mentions. For each adverse event within each response group, we compared the prevalence in tirzepatide versus semaglutide cohorts using χ^2^ tests for adequate cell counts (≥5 expected events per cell) or Fisher's exact test for smaller counts. We performed 16 independent tests within the high response, moderate response, and minimal weight-loss categories separately and reported unadjusted *P*-values at *P* < 0.05 (Fig. 2).

## Results

### Comparison of magnitude and rate of weight reduction in patients treated with tirzepatide versus semaglutide

From initial cohorts of 34,143 and 16,034 patients with at least three prescriptions for semaglutide or tirzepatide, respectively, we created 1:1 propensity-matched cohorts of 10,339 patients each (see Methods). The standardized mean differences for all variables used in matching were <0.1, indicative of adequate balance. Over 2 years of follow-up, tirzepatide was associated with markedly greater maximal weight reduction than semaglutide (Fig. 1, Tables S2–S4). The mean of maximal 2-year weight reduction was 14.7% (16.0 kg) for patients treated with tirzepatide compared with 10.8% (11.6 kg) for patients treated with semaglutide (*P* < 0.001, two-proportion *z*-test, Fig. 1a). Further, the proportion achieving their highest recorded weight loss of ≥5% at any time point was 90% with tirzepatide and 82% with semaglutide (*P* < 0.001). Differences widened at higher thresholds: 68 versus 47% achieved ≥10% loss (*P* < 0.001), and 43 versus 22% achieved ≥15% loss (*P* < 0.001), respectively (Fig. 1a–c). Maximal reductions of ≥20% were observed in 26% of tirzepatide-treated patients compared with 12% of semaglutide-treated patients (*P* < 0.001), and ≥25% reductions were observed in 14 versus 5% (*P* < 0.001; Fig. 1a–c). We compared the rate of weight loss between tirzepatide and semaglutide across all the weight-loss groups (Fig. 1d–h). During the first 6 months after treatment initiation, tirzepatide was associated with more rapid weight reduction than semaglutide in the high-response group (Fig. 1d). The mean monthly rate of weight loss was 2.54% for tirzepatide and 2.18% for semaglutide (*P* < 0.001) in the high-response group. Taken together, these findings indicate that compared with semaglutide tirzepatide produced overall higher weight loss and among high responders faster weight loss.

### Demographic patterns in weight-loss response in patients treated with tirzepatide versus semaglutide

We evaluated the weight-loss response distributions by race/ethnicity and sex for both tirzepatide and semaglutide (Table S2). White individuals were more represented in the high-response group than the minimal response group for both tirzepatide (91.2 vs. 79.8%, *P* < 0.001, two-proportion *z*-test) and semaglutide (91.2 vs. 79.1%, *P* < 0.001). On the contrary, Black patients were more represented in the minimal weight-loss group than the high-response group for both tirzepatide (10.9 vs. 6.0%, *P* < 0.001) and semaglutide (10.6 vs. 5.8%, *P* < 0.001), with a similar pattern observed among Hispanic patients for tirzepatide (3.3 vs. 1.6%, *P* < 0.001) and semaglutide (4.1 vs. 1.3%, *P* < 0.001). Evaluating response distribution by sex, females were more represented in the high-response group than the minimal response group for both tirzepatide (74.9 vs. 59.2%, *P* < 0.001) and semaglutide (80.3 vs. 58.5%, *P* < 0.001).

### Comparison of the prevalence of adverse events in patients treated with tirzepatide versus semaglutide

We compared the prevalence of 16 commonly reported adverse events between response-group–specific propensity-matched cohorts. The baseline and follow-up characteristics of the cohorts are summarized in Tables S5–S7. In the high-response group, the posttreatment prevalence of multiple gastrointestinal phenotypes was significantly lower among patients treated with tirzepatide compared with semaglutide, including nausea (27.2 vs. 31.4%, *P* = 0.002), vomiting (12.8 vs. 17.1%, *P* < 0.001), constipation (16.9 vs. 20.2%, *P* = 0.005), diarrhea (16.9 vs. 19.2%, *P* = 0.04), and dyspepsia (0.7 vs. 1.6%, *P* = 0.006; Table S8). Additionally, tirzepatide-treated patients in the high-response group had lower prevalence of fatigue (26.1 vs. 29.7%, *P* = 0.007) and headache (23.5 vs. 28.4%, *P* < 0.001; Table S8). In the moderate-response groups (*n* = 2,962), tirzepatide was also associated with a lower posttreatment prevalence of nausea (16.4 vs. 19.4%, *P* = 0.003), vomiting (7.8 vs. 9.6%, *P* = 0.02), and dizziness (8.9 vs. 11.0%, *P* = 0.007; Table S8).

## Discussion

In this large, propensity-matched real-world comparative analysis of tirzepatide and semaglutide, tirzepatide was associated with greater weight loss overall and faster weight-loss trajectories, with nearly twice the proportion of patients achieving ≥15% reductions. These findings extend observations from randomized trials (3, 14) and other real-world studies (15, 16) in highlighting diversity in weight-loss outcomes. Our findings underscore the profound heterogeneity of real-world outcomes: even after controlling for baseline characteristics, patients exhibited a wide distribution ranging from minimal change to >25% loss. Such dispersion illustrates the limitations of population-level efficacy metrics and the need for individualized treatment frameworks.

Demographics differences in sex and race/ethnicity associated with weight-loss response were mirrored across both tirzepatide and semaglutide, suggesting that the observed distribution is not specific to a single agent. Although the underlying contributors to these differences cannot be determined from observational data, potential factors may include variation in baseline clinical characteristics, access to care, treatment persistence, dose escalation, social determinants of health, and unmeasured biological heterogeneity. The consistency of these response distributions across two distinct GLP-1RA therapies underscores the importance of further investigation into the multidimensional factors that shape weight-loss outcomes in diverse populations.

Differences between tirzepatide and semaglutide extended beyond weight loss. Patients treated with tirzepatide demonstrated consistently lower posttreatment prevalence of gastrointestinal and systemic adverse events across response strata, despite achieving higher magnitude and faster weight reductions. This dissociation suggests that tolerability may depend not solely on total weight loss or dose intensity, but on mechanistic distinctions between dual incretin agonism and GLP-1–only therapy. Improved tolerability among high responders may facilitate sustained adherence, contributing to the broader response distribution observed for tirzepatide.

This study has several limitations. First, it is a retrospective observational analysis and therefore is prone to multiple sources of confounding and bias. Propensity matching addresses this concern to some extent, but it is likely that there are remaining unmeasured sources of confounding, including but not limited to concurrent use of other medications that can impact weight trajectories such as metformin and insulin, differences in treatment adherence, and documentation bias. Of note, the proportion of patients who had a documented order or administration of insulin or other diabetic medication classes was similar between the response-group–specific propensity-matched cohorts (Table S9). Second, race, insurance status, and other social determinants of health were not accounted for in the study design. Race itself is not known to be associated with GLP-1RA response and has historically not been included as a covariate in real-world analyses of GLP-1RA initiation or response (17, 18). That said, it is possible that there are differences in the degree of sustained access to care among populations of different racial and/or ethnic backgrounds despite the inclusion criteria that ensure the presence of weight measurements during multiple pre- and posttreatment intervals. Insurance status is associated with the likelihood of therapy initiation and continuation, but this information is not uniformly available in the dataset analyzed in this study. Third, this study did not assess dose escalation trajectories, including maximum dose achieved and duration of therapy, which have been shown to impact weight-loss magnitude in randomized trials and real-world studies from our group and others (3, 18, 19). While prescribed doses can be extracted from EHR prescription data, true dose escalation patterns (e.g. exact start dates, dates of dose increases, temporary treatment pauses, or dose de-escalations) are incompletely captured and are thus difficult to assess in real-world observational studies. Fourth, treatment effectiveness and documented adverse events were assessed independently, but it is likely that these are related to each other in the real-world setting. For example, it is possible that the lower rate of documented gastrointestinal and systemic adverse events among patients taking tirzepatide could contribute to higher observed effectiveness or that higher effectiveness reduces the likelihood of adverse events reporting. This probable interaction of effectiveness and tolerability make it difficult to disentangle cause and effect in our study framework. Future studies that account for treatment discontinuation, re-initiation, switching, and/or dose de-escalation could help to address this shortcoming. Finally, the different formulations of semaglutide (e.g. Ozempic and Wegovy) and tirzepatide (e.g. Mounjaro and Zepbound) were grouped together in this study. While the molecules are indeed the same in these formulations, there is variability in the recommended doses, approved clinical indications, and typical clinical use cases, all of which could impact the findings presented here.

In summary, tirzepatide was associated with greater and faster weight loss, lower adverse-event prevalence, and larger reductions in multiple disease burdens compared with semaglutide, while both agents produced meaningful improvements across metabolic, respiratory, and neuropsychiatric domains. The wide spectrum of outcomes and demographic gradients observed emphasize the urgent need for precision approaches to obesity treatment and highlight opportunities for next-generation incretin therapeutics informed by real-world response phenotypes.

## Supplementary Material

pgag171_Supplementary_Data

## Data Availability

This study involves the analysis of de-identified EHR data via the nference nSights Federated Clinical Analytics Platform (nSights). Data shown and reported in this manuscript were extracted from this environment using an established protocol for data extraction, aimed at preserving patient privacy. The data have been de-identified pursuant to an expert determination in accordance with the HIPAA Privacy Rule. Any data beyond what is reported in the manuscript, including but not limited to the raw EHR data, cannot be shared or released due to the parameters of the expert determination to maintain the data de-identification. Contact the corresponding author for additional details regarding nSights.

## Preprints

This manuscript was posted as a preprint at https://www.medrxiv.org/content/10.64898/2025.11.30.25341294v1.
