# Supplementary material for: Weight-loss dynamics with tirzepatide versus semaglutide
Source: PNAS Nexus. 2026 Jun 16;5(6):pgag171. doi: 10.1093/pnasnexus/pgag171 (PMC13270487; doi:10.1093/pnasnexus/pgag171)
Supplement: pgag171_Supplementary_Data [file pgag171_supplementary_data.docx]

### **Supplementary Material**


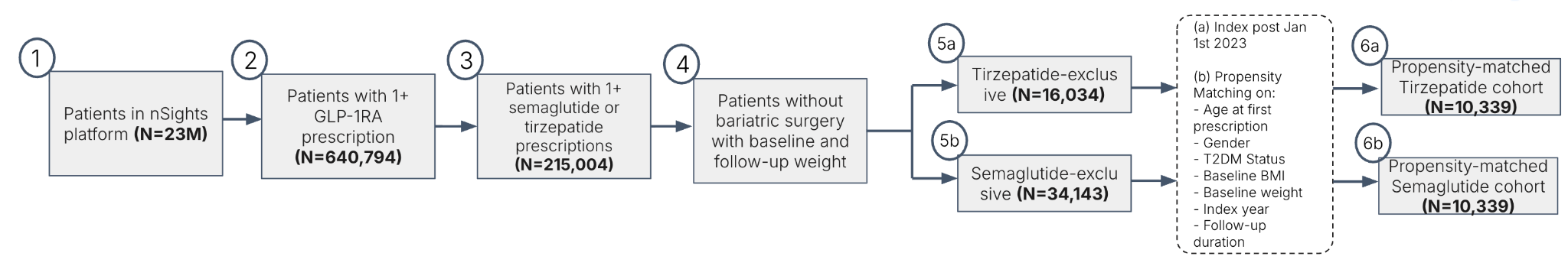


**Figure S1: Cohort Funnel based on inclusion and exclusion criteria**

**
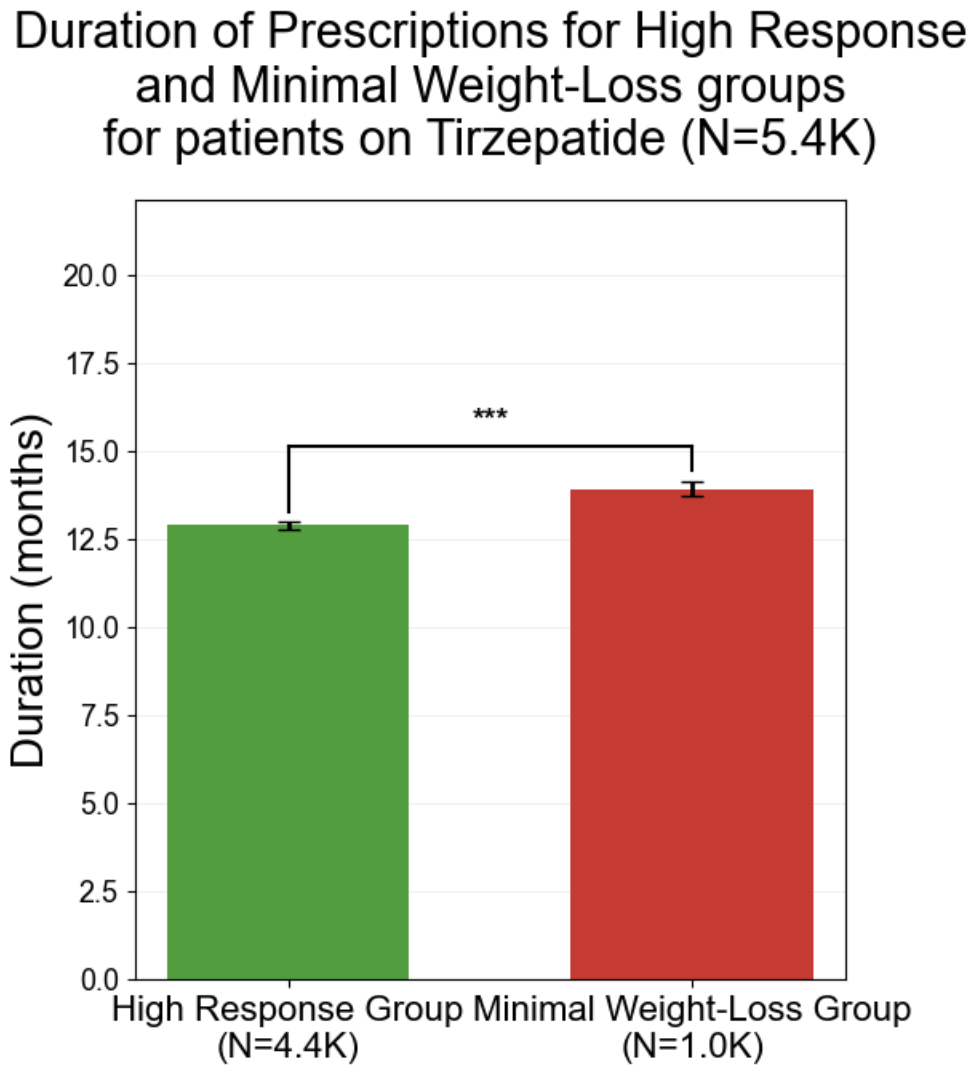

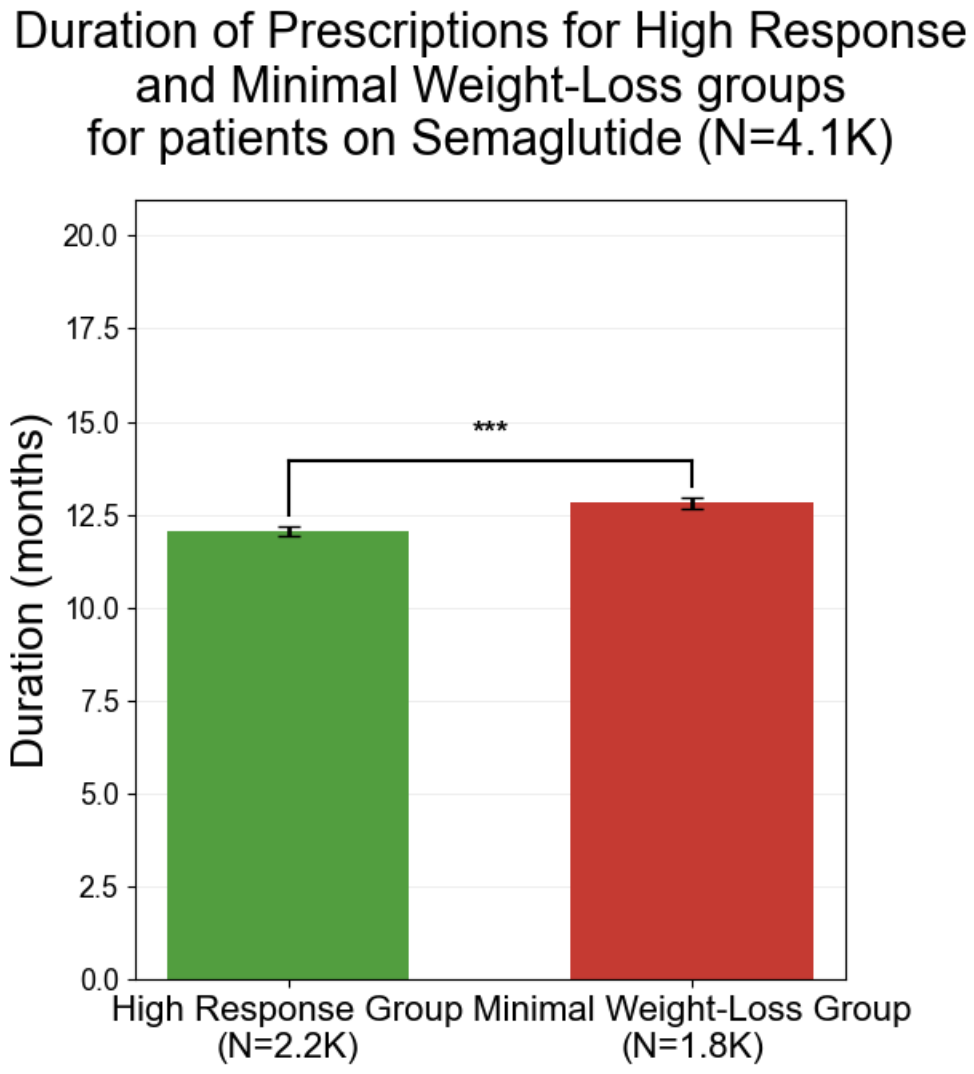
**

**Figure S2: Distribution of duration of prescriptions (time from first to last prescription) in patients treated with (a) tirzepatide and (b) semaglutid**e

**Table S1. Brand name distribution among the overall propensity-matched cohorts of tirzepatide-treated and semaglutide-treated patients (N=10,339).** For each active compound (tirzepatide or semaglutide), the number of patients with at least one prescription for a given brand name formulation or combination of brand name formulations is provided.

| **Active Compound** | **Brand name / Combination** | **Number of patients** |
| --- | --- | --- |
| Tirzepatide | Mounjaro | 5,829 |
|  | Zepbound | 2,194 |
|  | Mounjaro & Zepbound | 2,316 |
| Semaglutide | Ozempic | 5,851 |
|  | Wegovy | 2,862 |
|  | Ozempic & Wegovy | 850 |
|  | Rybelsus | 429 |
|  | Ozempic & Rybelsus | 294 |
|  | Wegovy & Rybelsus | 35 |
|  | Ozempic & Wegovy & Rybelsus | 18 |

**Table S2. Demographic characteristics of the overall propensity-matched cohorts of tirzepatide-treated and semaglutide-treated cohorts (N = 10,339).** The summary characteristics are provided for each weight loss outcome category (high response, moderate response, late response, weight regain, and minimal weight loss). The brand name formulations for semaglutide included Ozempic, Wegovy, and Rybelsus. The brand name formulations for tirzepatide included Mounjaro and Zepbound.

| **Weight Loss Category** | **Characteristic** | **Semaglutide**  **(Overall N = 10,339)** | **Tirzepatide**  **(Overall N = 10,339)** |
| --- | --- | --- | --- |
| High Response | Total, N (% of Total) | 2235 (21.6%) | 4402 (42.6%) |
|  | Male, N (% of Group) | 439 (19.64%) | 1108 (25.17%) |
|  | Age, Mean (SD) | 51.80 (14.93) | 52.14 (13.26) |
|  | Age, Median [IQR] | 51.47 [40.66, 63.37] | 52.38 [42.69, 62.28] |
|  | Race, N (% of Group)   - White - Black - Hispanic - Other / Unknown | - 2039 (91.2%) - 130 (5.8%) - 29 (1.3%) - 108 (4.8%) | - 4014 (91.2%) - 265 (6.0%) - 70 (1.6%) - 162 (3.7%) |
|  |  | | |
| Moderate Response | Total, N (% of Total) | 5006 (48.4%) | 3981 (38.5%) |
|  | Male, N (% of Group) | 1925 (38.45%) | 1666 (41.85%) |
|  | Age, Mean (SD) | 56.08 (14.36) | 55.09 (13.38) |
|  | Age, Median [IQR] | 57.25 [46.66, 66.83] | 55.87 [45.46, 64.93] |
|  | Race, N (% of Group)   - White - Black - Hispanic - Other / Unknown | - 4371 (87.3%) - 382 (7.6%) - 125 (2.5%) - 252 (5.0%) | - 3480 (87.4%) - 272 (6.8%) - 81 (2.0%) - 227 (5.7%) |
|  |  | | |
| Weight Regain | Total, N (% of Total) | 499 (4.8%) | 429 (4.1%) |
|  | Male, N (% of Group) | 177 (35.47%) | 142 (33.1%) |
|  | Age, Mean (SD) | 52.64 (15.18) | 52.25 (13.88) |
|  | Age, Median [IQR] | 53.39 [40.60, 63.75] | 52.93 [42.47, 62.24] |
|  | Race, N (% of Group)   - White - Black - Hispanic - Other / Unknown | - 427 (85.6%) - 48 (9.6%) - 8 (1.6%) - 23 (4.6%) | - 378 (88.1%) - 22 (5.1%) - 16 (3.7%) - 18 (4.2%) |
|  |  | | |
| Late Response | Total, N (% of Total) | 755 (7.3%) | 526 (5.1%) |
|  | Male, N (% of Group) | 281 (37.22%) | 194 (36.88%) |
|  | Age, Mean (SD) | 53.40 (13.99) | 55.28 (12.61) |
|  | Age, Median [IQR] | 54.34 [43.65, 64.07] | 55.98 [47.03, 64.28] |
|  | Race, N (% of Group)   - White - Black - Hispanic - Other / Unknown | - 634 (84.0%) - 60 (7.9%) - 24 (3.2%) - 50 (6.6%) | - 457 (86.9%) - 35 (6.7%) - 14 (2.7%) - 24 (4.6%) |
|  |  | | |
| Minimal Response | Total, N (% of Total) | 1844 (17.8%) | 1001 (9.7%) |
|  | Male, N (% of Group) | 766 (41.54%) | 408 (40.76%) |
|  | Age, Mean (SD) | 52.64 (14.52) | 52.56 (13.20) |
|  | Age, Median [IQR] | 53.69 [41.83, 64.28] | 53.03 [43.04, 62.23] |
|  | Race, N (% of Group)   - White - Black - Hispanic - Other / Unknown | - 1458 (79.1%) - 196 (10.6%) - 76 (4.1%) - 137 (7.4%) | - 799 (79.8%) - 109 (10.9%) - 33 (3.3%) - 66 (6.6%) |

**Table S3. Baseline clinical characteristics and data availability of the overall propensity-matched cohorts of tirzepatide-treated and semaglutide-treated cohorts (N = 10,339).** The characteristics are provided for each weight loss outcome category (high response, moderate response, late response, weight regain, and minimal weight loss). The brand name formulations for semaglutide included Ozempic, Wegovy, and Rybelsus. The brand name formulations for tirzepatide included Mounjaro and Zepbound.

| **Weight Loss Category** | **Characteristic** | **Semaglutide**  **(Overall N = 10,339)** | **Tirzepatide**  **(Overall N = 10,339)** |
| --- | --- | --- | --- |
| High Response | Total, N (% of Total) | 2235 (21.6%) | 4402 (42.6%) |
|  | Type 2 Diabetes, N (% of Group) | 477 (21.34%) | 1072 (24.35%) |
|  | Baseline BMI, Mean (SD) | 37.01 (6.09) | 37.53 (6.42) |
|  | Baseline BMI, Median [IQR] | 36.39 [32.42, 41.71] | 36.94 [32.78, 41.95] |
|  | Baseline Weight in kg, Mean (SD) | 104.54 (22.19) | 107.39 (22.75) |
|  | Baseline Weight in kg, Median [IQR] | 101.64 [88.47, 117.71] | 104.07 [90.54, 121.36] |
|  | Pre-GLP-1RA Weight Measurements, Mean (SD) | 33.91 (32.68) | 31.15 (30.92) |
|  | Pre-GLP-1RA Weight Measurements, Median [IQR] | 25 [11, 45] | 23 [11, 42] |
|  | Baseline Monthly Documents Per Patient, Mean (SD) | 6.3 (14.0) | 6.0 (13.4) |
|  | Baseline Monthly Documents Per Patient, Median [IQR] | 3 [2, 6] | 3 [1, 6] |
|  |  | | |
| Moderate Response | Total, N (% of Total) | 5006 (48.4%) | 3981 (38.5%) |
|  | Type 2 Diabetes, N (% of Group) | 1713 (34.22%) | 1362 (34.21%) |
|  | Baseline BMI, Mean (SD) | 36.90 (6.62) | 36.88 (6.69) |
|  | Baseline BMI, Median [IQR] | 36.40 [32.15, 41.38] | 36.34 [32.06, 41.27] |
|  | Baseline Weight in kg, Mean (SD) | 107.52 (23.37) | 108.52 (24.09) |
|  | Baseline Weight in kg, Median [IQR] | 104.64 [90.53, 122.20] | 106.16 [91.24, 123.01] |
|  | Pre-GLP-1RA Weight Measurements, Mean (SD) | 34.61 (42.25) | 30.72 (29.47) |
|  | Pre-GLP-1RA Weight Measurements, Median [IQR] | 25 [11, 46] | 23 [11, 42] |
|  | Baseline Monthly Documents Per Patient, Mean (SD) | 6.5 (14.0) | 6.1 (13.7) |
|  | Baseline Monthly Documents Per Patient, Median [IQR] | 3 [2, 6] | 3 [1, 6] |
|  |  | | |
| Weight Regain | Total, N (% of Total) | 499 (4.8%) | 429 (4.1%) |
|  | Type 2 Diabetes, N (% of Group) | 150 (30.06%) | 122 (28.44%) |
|  | Baseline BMI, Mean (SD) | 36.02 (6.84) | 35.59 (6.34) |
|  | Baseline BMI, Median [IQR] | 35.25 [31.14, 41.47] | 34.82 [31.10, 39.06] |
|  | Baseline Weight in kg, Mean (SD) | 104.70 (24.69) | 101.79 (22.23) |
|  | Baseline Weight in kg, Median [IQR] | 100.83 [86.87, 119.88] | 97.82 [86.46, 115.09] |
|  | Pre-GLP-1RA Weight Measurements, Mean (SD) | 36.63 (37.39) | 30.70 (29.00) |
|  | Pre-GLP-1RA Weight Measurements, Median [IQR] | 26 [12, 49] | 24 [10, 39] |
|  | Baseline Monthly Documents Per Patient, Mean (SD) | 8.3 (19.6) | 6.6 (12.9) |
|  | Baseline Monthly Documents Per Patient, Median [IQR] | 3 [2, 7] | 3 [1, 7] |
|  |  | | |
| Late Response | Total, N (% of Total) | 755 (7.3%) | 526 (5.1%) |
|  | Type 2 Diabetes, N (% of Group) | 241 (31.92%) | 177 (33.65%) |
|  | Baseline BMI, Mean (SD) | 37.91 (7.33) | 38.03 (7.38) |
|  | Baseline BMI, Median [IQR] | 37.05 [32.59, 42.07] | 37.59 [33.07, 41.92] |
|  | Baseline Weight in kg, Mean (SD) | 110.44 (24.44) | 111.70 (24.67) |
|  | Baseline Weight in kg, Median [IQR] | 108.76 [92.61, 125.46] | 107.97 [93.07, 126.87] |
|  | Pre-GLP-1RA Weight Measurements, Mean (SD) | 29.36 (27.97) | 28.84 (29.48) |
|  | Pre-GLP-1RA Weight Measurements, Median [IQR] | 22 [11, 39] | 21 [11, 37] |
|  | Baseline Monthly Documents Per Patient, Mean (SD) | 6.6 (13.5) | 6.8 (14.6) |
|  | Baseline Monthly Documents Per Patient, Median [IQR] | 3 [1, 7] | 3 [1, 6] |
|  |  | | |
| Minimal Response | Total, N (% of Total) | 1844 (17.8%) | 1001 (9.7%) |
|  | Type 2 Diabetes, N (% of Group) | 568 (30.8%) | 272 (27.17%) |
|  | Baseline BMI, Mean (SD) | 37.63 (7.63) | 38.23 (9.18) |
|  | Baseline BMI, Median [IQR] | 36.80 [32.39, 42.13] | 37.48 [31.51, 43.64] |
|  | Baseline Weight in kg, Mean (SD) | 110.60 (26.10) | 112.69 (29.68) |
|  | Baseline Weight in kg, Median [IQR] | 107.32 [92.36, 126.10] | 109.51 [91.49, 131.97] |
|  | Pre-GLP-1RA Weight Measurements, Mean (SD) | 29.31 (30.23) | 25.82 (23.57) |
|  | Pre-GLP-1RA Weight Measurements, Median [IQR] | 21 [9, 39] | 20 [9, 35] |
|  | Baseline Monthly Documents Per Patient, Mean (SD) | 7.0 (15.3) | 6.5 (13.2) |
|  | Baseline Monthly Documents Per Patient, Median [IQR] | 3 [1, 7] | 3 [1, 6] |

**Table S4. Post-treatment characteristics in the overall propensity-matched cohorts of tirzepatide-treated and semaglutide-treated cohorts (N = 10,339).** The summary characteristics are provided for each weight loss outcome category (high response, moderate response, late response, weight regain, and minimal weight loss). The brand name formulations for semaglutide included Ozempic, Wegovy, and Rybelsus. The brand name formulations for tirzepatide included Mounjaro and Zepbound.

| **Weight Loss Category** | **Characteristic** | **Summary Statistic** | **Semaglutide**  **(Overall N = 10,339)** | **Tirzepatide**  **(Overall N = 10,339)** |
| --- | --- | --- | --- | --- |
| High Response | Duration of prescriptions per patient in months | Mean (SD) | 12.04 (6.52) | 12.90 (6.12) |
|  |  | Median [IQR] | 11.46 [7.11, 16.56] | 12.18 [8.60, 16.95] |
|  | Number of prescriptions per patient | Mean (SD) | 12.25 (8.53) | 16.82 (11.16) |
|  |  | Median [IQR] | 10 [6, 16] | 15 [9, 22] |
|  | Follow-up duration in days, Mean (SD) | Mean (SD) | 501.46 (198.68) | 491.06 (187.27) |
|  |  | Median [IQR] | 500 [354, 637] | 453 [352, 625] |
|  | Number of follow-up events per patient | Mean (SD) | 805.1 (1843.7) | 617.8 (1349.5) |
|  |  | Median [IQR] | 356 [201, 718] | 305 [181, 585] |
|  | Post-GLP1RA Weight Measurements | Mean (SD) | 10.84 (12.09) | 9.44 (9.19) |
|  |  | Median [IQR] | 8 [5, 13] | 7 [5, 11] |
|  | Post-treatment monthly documents per patient | Mean (SD) | 6.0 (14.6) | 4.9 (9.1) |
|  |  | Median [IQR] | 3 [2, 6] | 3 [2, 5] |
|  | | | | |
| Moderate Response | Duration of prescriptions per patient in months | Mean (SD) | 9.45 (5.93) | 11.27 (6.09) |
|  |  | Median [IQR] | 8.19 [4.99, 12.71] | 10.29 [7.03, 14.62] |
|  | Number of prescriptions per patient | Mean (SD) | 9.50 (6.35) | 13.51 (9.24) |
|  |  | Median [IQR] | 8 [5, 12] | 12 [7, 17] |
|  | Follow-up duration in days, Mean (SD) | Mean (SD) | 409.44 (194.60) | 441.70 (183.43) |
|  |  | Median [IQR] | 375 [266, 531] | 399 [306, 560] |
|  | Number of follow-up events per patient | Mean (SD) | 565.1 (1052.7) | 504.4 (957.7) |
|  |  | Median [IQR] | 290 [163, 553] | 276 [164, 504] |
|  | Post-GLP1RA Weight Measurements | Mean (SD) | 8.47 (7.31) | 8.06 (7.10) |
|  |  | Median [IQR] | 6 [4, 10] | 6 [4, 9] |
|  | Post-treatment monthly documents per patient | Mean (SD) | 5.7 (10.5) | 5.0 (8.4) |
|  |  | Median [IQR] | 3 [2, 6] | 3 [2, 6] |
|  | | | | |
| Weight Regain | Duration of prescriptions per patient in months | Mean (SD) | 11.31 (6.77) | 12.97 (7.07) |
|  |  | Median [IQR] | 11.47 [5.61, 15.83] | 13.13 [6.86, 18.14] |
|  | Number of prescriptions per patient | Mean (SD) | 9.79 (6.99) | 13.62 (9.88) |
|  |  | Median [IQR] | 8 [5, 12] | 11 [6, 19] |
|  | Follow-up duration in days, Mean (SD) | Mean (SD) | 570.41 (144.15) | 580.63 (147.98) |
|  |  | Median [IQR] | 537 [457, 674] | 552 [457, 693] |
|  | Number of follow-up events per patient | Mean (SD) | 1233.7 (2499.9) | 969.2 (2254.7) |
|  |  | Median [IQR] | 499 [244, 989] | 468 [268, 928] |
|  | Post-GLP1RA Weight Measurements | Mean (SD) | 14.00 (12.74) | 13.16 (11.65) |
|  |  | Median [IQR] | 10 [7, 16] | 10 [7, 16] |
|  | Post-treatment monthly documents per patient | Mean (SD) | 8.3 (20.0) | 6.6 (13.6) |
|  |  | Median [IQR] | 4 [2, 8] | 4 [2, 7] |
|  | | | | |
| Late Response | Duration of prescriptions per patient in months | Mean (SD) | 14.73 (6.71) | 15.90 (6.66) |
|  |  | Median [IQR] | 15.38 [10.09, 19.84] | 16.69 [12.54, 20.44] |
|  | Number of prescriptions per patient | Mean (SD) | 10.80 (7.34) | 13.34 (9.52) |
|  |  | Median [IQR] | 9 [6, 14] | 12 [6, 18] |
|  | Follow-up duration in days, Mean (SD) | Mean (SD) | 630.48 (127.45) | 631.41 (137.83) |
|  |  | Median [IQR] | 629 [543, 724] | 630 [528, 732] |
|  | Number of follow-up events per patient | Mean (SD) | 801.0 (1688.2) | 679.2 (1098.7) |
|  |  | Median [IQR] | 376 [240, 693] | 393 [242, 715] |
|  | Post-GLP1RA Weight Measurements | Mean (SD) | 11.13 (9.48) | 10.29 (8.05) |
|  |  | Median [IQR] | 8 [6, 13] | 8 [6, 12] |
|  | Post-treatment monthly documents per patient | Mean (SD) | 4.8 (7.1) | 4.2 (5.7) |
|  |  | Median [IQR] | 3 [1, 6] | 3 [1, 5] |
|  | | | | |
| Minimal Response | Duration of prescriptions per patient in months | Mean (SD) | 12.81 (6.57) | 13.94 (6.94) |
|  |  | Median [IQR] | 12.98 [7.92, 17.28] | 14.05 [9.13, 18.60] |
|  | Number of prescriptions per patient | Mean (SD) | 8.85 (6.23) | 11.02 (8.63) |
|  |  | Median [IQR] | 8 [4, 12] | 8 [4, 15] |
|  | Follow-up duration in days, Mean (SD) | Mean (SD) | 585.78 (135.60) | 578.62 (142.68) |
|  |  | Median [IQR] | 572 [474, 691] | 558 [463, 683] |
|  | Number of follow-up events per patient | Mean (SD) | 608.8 (1168.9) | 526.5 (978.5) |
|  |  | Median [IQR] | 335 [212, 574] | 314 [207, 530] |
|  | Post-GLP1RA Weight Measurements | Mean (SD) | 9.94 (7.46) | 8.84 (6.42) |
|  |  | Median [IQR] | 8 [5, 12] | 7 [5, 11] |
|  | Post-treatment monthly documents per patient | Mean (SD) | 5.2 (8.5) | 4.8 (7.6) |
|  |  | Median [IQR] | 3 [2, 6] | 3 [1, 6] |

**Table S5. Demographic characteristics of the response-group-specific propensity-matched cohorts of tirzepatide-treated versus semaglutide-treated cohorts.** For these cohorts, 1:1 propensity matching was performed within each response group, yielding different subpopulation sizes for each group: high response (N = 2318), moderate response (N = 2692), weight regain (N = 285), late response (N = 293), and minimal response (N = 540). The summary characteristics are provided for each of these weight loss outcome categories. The brand name formulations for semaglutide included Ozempic, Wegovy, and Rybelsus. The brand name formulations for tirzepatide included Mounjaro and Zepbound.

| **Weight Loss Category** | **Characteristic** | **Semaglutide** | **Tirzepatide** |
| --- | --- | --- | --- |
| High Response  (N = 2318) | Male, N (% of Group) | 531 (22.91%) | 576 (24.85%) |
|  | Age, Mean (SD) | 53.52 (15.12) | 53.15 (13.31) |
|  | Age, Median [IQR] | 54.16 [42.33, 65.23] | 53.23 [43.67, 63.23] |
|  | Race, N (% of Group)   - White - Black - Hispanic - Other / Unknown | - 2160 (93.2%) - 128 (5.5%) - 28 (1.2%) - 91 (3.9%) | - 2148 (92.7%) - 133 (5.7%) - 38 (1.6%) - 81 (3.5%) |
|  |  | | |
| Moderate Response  (N = 2692) | Male, N (% of Group) | 1200 (40.51%) | 1190 (40.18%) |
|  | Age, Mean (SD) | 55.77 (14.50) | 55.50 (13.68) |
|  | Age, Median [IQR] | 57.19 [45.83, 66.88] | 56.62 [45.41, 65.68] |
|  | Race, N (% of Group)   - White - Black - Hispanic - Other / Unknown | - 2611 (88.1%) - 226 (7.6%) - 86 (2.9%) - 137 (4.6%) | - 2643 (89.2%) - 193 (6.5%) - 48 (1.6%) - 153 (5.2%) |
|  |  | | |
| Weight Regain  (N = 285) | Male, N (% of Group) | 99 (34.74%) | 96 (33.68%) |
|  | Age, Mean (SD) | 52.64 (15.01) | 53.06 (14.06) |
|  | Age, Median [IQR] | 53.48 [39.99, 64.52] | 53.90 [43.02, 62.80] |
|  | Race, N (% of Group)   - White - Black - Hispanic - Other / Unknown | - 248 (87.0%) - 20 (7.0%) - 3 (1.1%) - 19 (6.7%) | - 254 (89.1%) - 13 (4.6%) - 10 (3.5%) - 13 (4.6%) |
|  |  | | |
| Late Response  (N = 293) | Male, N (% of Group) | 106 (35.33%) | 110 (36.67%) |
|  | Age, Mean (SD) | 54.66 (15.24) | 55.34 (12.61) |
|  | Age, Median [IQR] | 57.47 [43.98, 66.20] | 56.21 [48.12, 64.21] |
|  | Race, N (% of Group)   - White - Black - Hispanic - Other / Unknown | - 258 (86.0%) - 25 (8.3%) - 6 (2.0%) - 21 (7.0%) | - 257 (85.7%) - 25 (8.3%) - 7 (2.3%) - 15 (5.0%) |
|  |  | | |
| Minimal Response  (N = 540) | Male, N (% of Group) | 218 (40.37%) | 221 (40.93%) |
|  | Age, Mean (SD) | 52.84 (14.53) | 52.89 (13.87) |
|  | Age, Median [IQR] | 54.11 [41.48, 64.24] | 53.70 [42.90, 63.21] |
|  | Race, N (% of Group)   - White - Black - Hispanic - Other / Unknown | - 433 (80.2%) - 65 (12.0%) - 18 (3.3%) - 36 (6.7%) | - 442 (81.9%) - 53 (9.8%) - 14 (2.6%) - 35 (6.5%) |

**Table S6. Baseline clinical characteristics and data availability of the response-group-specific propensity-matched cohorts of tirzepatide-treated versus semaglutide-treated cohorts.** For these cohorts, 1:1 propensity matching was performed within each response group, yielding different subpopulation sizes for each group: high response (N = 2318), moderate response (N = 2692), weight regain (N = 285), late response (N = 293), and minimal response (N = 540). The summary characteristics are provided for each of these weight loss outcome categories. The brand name formulations for semaglutide included Ozempic, Wegovy, and Rybelsus. The brand name formulations for tirzepatide included Mounjaro and Zepbound.

| **Weight Loss Category** | **Characteristic** | **Semaglutide** | **Tirzepatide** |
| --- | --- | --- | --- |
| High Response  (N = 2318) | Type 2 Diabetes, N (%) | 621 (26.79%) | 658 (28.39%) |
|  | Baseline BMI, Mean (SD) | 36.93 (5.94) | 37.52 (6.30) |
|  | Baseline BMI, Median [IQR] | 36.44 [32.56, 41.55] | 37.18 [32.82, 42.13] |
|  | Baseline Weight in kg, Mean (SD) | 105.21 (22.31) | 108.19 (23.13) |
|  | Baseline Weight in kg, Median [IQR] | 101.79 [88.47, 118.73] | 104.96 [90.59, 122.66] |
|  | Pre-GLP-1RA Weight Measurements, Mean (SD) | 38.18 (37.67) | 34.29 (34.46) |
|  | Pre-GLP-1RA Weight Measurements, Median [IQR] | 27 [13, 50] | 25 [13, 45] |
|  | Baseline Monthly Documents Per Patient, Mean (SD) | 6.7 (15.4) | 6.2 (14.6) |
|  | Baseline Monthly Documents Per Patient, Median [IQR] | 3 [2, 6] | 3 [2, 6] |
|  | | | |
| Moderate Response  (N = 2692) | Type 2 Diabetes, N (%) | 1015 (34.27%) | 1003 (33.86%) |
|  | Baseline BMI, Mean (SD) | 36.78 (6.47) | 36.93 (6.47) |
|  | Baseline BMI, Median [IQR] | 36.34 [32.14, 41.31] | 36.57 [32.23, 41.40] |
|  | Baseline Weight in kg, Mean (SD) | 108.14 (23.76) | 108.97 (24.09) |
|  | Baseline Weight in kg, Median [IQR] | 105.37 [90.77, 123.11] | 106.49 [91.21, 123.34] |
|  | Pre-GLP-1RA Weight Measurements, Mean (SD) | 37.87 (37.14) | 34.15 (32.20) |
|  | Pre-GLP-1RA Weight Measurements, Median [IQR] | 28 [12, 52] | 26 [12, 46] |
|  | Baseline Monthly Documents Per Patient, Mean (SD) | 6.8 (15.2) | 6.2 (13.5) |
|  | Baseline Monthly Documents Per Patient, Median [IQR] | 3 [2, 7] | 3 [2, 6] |
|  | | | |
| Weight Regain  (N = 285) | Type 2 Diabetes, N (%) | 85 (29.82%) | 88 (30.88%) |
|  | Baseline BMI, Mean (SD) | 35.60 (6.76) | 35.39 (6.22) |
|  | Baseline BMI, Median [IQR] | 34.17 [30.90, 40.07] | 34.72 [31.07, 38.78] |
|  | Baseline Weight in kg, Mean (SD) | 101.72 (21.30) | 101.46 (22.04) |
|  | Baseline Weight in kg, Median [IQR] | 99.86 [86.17, 112.62] | 97.83 [86.50, 114.88] |
|  | Pre-GLP-1RA Weight Measurements, Mean (SD) | 36.44 (36.88) | 34.37 (31.73) |
|  | Pre-GLP-1RA Weight Measurements, Median [IQR] | 27 [13, 48] | 28 [13, 43] |
|  | Baseline Monthly Documents Per Patient, Mean (SD) | 8.0 (19.0) | 6.8 (13.3) |
|  | Baseline Monthly Documents Per Patient, Median [IQR] | 3 [2, 8] | 3 [2, 7] |
|  | | | |
| Late Response  (N = 293) | Type 2 Diabetes, N (%) | 104 (34.67%) | 107 (35.67%) |
|  | Baseline BMI, Mean (SD) | 37.91 (7.31) | 37.61 (6.77) |
|  | Baseline BMI, Median [IQR] | 37.52 [33.02, 42.16] | 37.37 [32.93, 41.70] |
|  | Baseline Weight in kg, Mean (SD) | 111.71 (24.09) | 111.40 (24.88) |
|  | Baseline Weight in kg, Median [IQR] | 108.87 [93.87, 126.39] | 107.39 [92.83, 126.81] |
|  | Pre-GLP-1RA Weight Measurements, Mean (SD) | 32.18 (29.91) | 32.16 (33.37) |
|  | Pre-GLP-1RA Weight Measurements, Median [IQR] | 26 [13, 42] | 23 [12, 44] |
|  | Baseline Monthly Documents Per Patient, Mean (SD) | 6.4 (13.5) | 7.2 (16.1) |
|  | Baseline Monthly Documents Per Patient, Median [IQR] | 3 [2, 6] | 3 [2, 7] |
|  | | | |
| Minimal Response  (N = 540) | Type 2 Diabetes, N (%) | 178 (32.96%) | 168 (31.11%) |
|  | Baseline BMI, Mean (SD) | 38.40 (7.99) | 37.97 (8.97) |
|  | Baseline BMI, Median [IQR] | 37.93 [32.42, 43.40] | 37.34 [31.48, 43.29] |
|  | Baseline Weight in kg, Mean (SD) | 113.66 (27.15) | 112.43 (29.37) |
|  | Baseline Weight in kg, Median [IQR] | 110.86 [93.15, 130.84] | 109.36 [91.52, 132.14] |
|  | Pre-GLP-1RA Weight Measurements, Mean (SD) | 33.49 (31.66) | 29.53 (26.27) |
|  | Pre-GLP-1RA Weight Measurements, Median [IQR] | 24 [12, 48] | 23 [10, 40] |
|  | Baseline Monthly Documents Per Patient, Mean (SD) | 7.3 (15.7) | 6.9 (14.0) |
|  | Baseline Monthly Documents Per Patient, Median [IQR] | 3 [2, 7] | 3 [2, 7] |

**Table S7. Post-treatment characteristics of the response-group-specific propensity-matched cohorts of tirzepatide-treated versus semaglutide-treated cohorts.** For these cohorts, 1:1 propensity matching was performed within each response group, yielding different subpopulation sizes for each group: high response (N = 2318), moderate response (N = 2692), weight regain (N = 285), late response (N = 293), and minimal response (N = 540). The summary characteristics are provided for each of these weight loss outcome categories. The brand name formulations for semaglutide included Ozempic, Wegovy, and Rybelsus. The brand name formulations for tirzepatide included Mounjaro and Zepbound.

| **Weight Loss Category** | **Characteristic** | **Summary Statistic** | **Semaglutide** | **Tirzepatide** |
| --- | --- | --- | --- | --- |
| High Response  (N = 2318) | Duration of prescriptions per patient in months | Mean (SD) | 12.50 (6.55) | 14.06 (6.02) |
|  |  | Median [IQR] | 12.00 [7.49, 17.10] | 13.51 [10.41, 18.04] |
|  | Number of prescriptions per patient | Mean (SD) | 12.67 (8.80) | 17.41 (11.53) |
|  |  | Median [IQR] | 11 [6, 16] | 16 [10, 22] |
|  | Follow-up duration in days, Mean (SD) | Mean (SD) | 517.68 (187.59) | 534.43 (178.91) |
|  |  | Median [IQR] | 517 [377, 646] | 516 [407, 647] |
|  | Number of follow-up events per patient | Mean (SD) | 961.3 (2249.5) | 741.3 (1564.0) |
|  |  | Median [IQR] | 415 [233, 860] | 376 [220, 718] |
|  | Post-GLP1RA Weight Measurements | Mean (SD) | 12.23 (11.97) | 10.88 (11.07) |
|  |  | Median [IQR] | 9 [6, 14] | 8 [5, 13] |
|  | Post-treatment monthly documents per patient | Mean (SD) | 6.1 (14.1) | 5.0 (8.7) |
|  |  | Median [IQR] | 3 [2, 6] | 3 [2, 6] |
| Moderate Response  (N = 2692) | Duration of prescriptions per patient in months | Mean (SD) | 9.16 (5.84) | 10.16 (5.94) |
|  |  | Median [IQR] | 7.80 [4.72, 12.57] | 9.04 [5.93, 13.25] |
|  | Number of prescriptions per patient | Mean (SD) | 9.61 (6.45) | 12.86 (8.73) |
|  |  | Median [IQR] | 8 [5, 13] | 11 [7, 17] |
|  | Follow-up duration in days, Mean (SD) | Mean (SD) | 394.35 (189.87) | 397.74 (185.78) |
|  |  | Median [IQR] | 356 [252, 515] | 356 [260, 502] |
|  | Number of follow-up events per patient | Mean (SD) | 619.4 (1235.8) | 531.1 (1012.9) |
|  |  | Median [IQR] | 299 [167, 609] | 282 [164, 537] |
|  | Post-GLP1RA Weight Measurements | Mean (SD) | 9.08 (9.21) | 8.32 (7.77) |
|  |  | Median [IQR] | 6 [5, 11] | 6 [4, 10] |
|  | Post-treatment monthly documents per patient | Mean (SD) | 5.8 (10.7) | 5.2 (8.8) |
|  |  | Median [IQR] | 3 [2, 6] | 3 [2, 6] |
| Weight Regain  (N = 285) | Duration of prescriptions per patient in months | Mean (SD) | 12.23 (6.95) | 12.73 (7.34) |
|  |  | Median [IQR] | 12.67 [6.83, 16.50] | 12.71 [6.62, 17.89] |
|  | Number of prescriptions per patient | Mean (SD) | 10.36 (7.62) | 13.53 (9.33) |
|  |  | Median [IQR] | 8 [6, 14] | 12 [6, 19] |
|  | Follow-up duration in days, Mean (SD) | Mean (SD) | 591.73 (150.19) | 583.86 (151.71) |
|  |  | Median [IQR] | 567 [470, 730] | 550 [459, 708] |
|  | Number of follow-up events per patient | Mean (SD) | 1104.8 (1869.9) | 1012.7 (2370.6) |
|  |  | Median [IQR] | 562 [282, 1083] | 500 [293, 943] |
|  | Post-GLP1RA Weight Measurements | Mean (SD) | 14.02 (10.30) | 13.70 (11.89) |
|  |  | Median [IQR] | 11 [7, 17] | 10 [7, 16] |
|  | Post-treatment monthly documents per patient | Mean (SD) | 7.6 (17.9) | 6.5 (12.0) |
|  |  | Median [IQR] | 4 [2, 7] | 3 [2, 7] |
| Late Response  (N = 293) | Duration of prescriptions per patient in months | Mean (SD) | 14.07 (6.90) | 15.82 (6.67) |
|  |  | Median [IQR] | 14.74 [8.70, 19.05] | 16.19 [12.59, 20.49] |
|  | Number of prescriptions per patient | Mean (SD) | 10.36 (7.26) | 13.15 (9.01) |
|  |  | Median [IQR] | 8 [5, 13] | 12 [6, 18] |
|  | Follow-up duration in days, Mean (SD) | Mean (SD) | 628.18 (126.36) | 626.63 (137.65) |
|  |  | Median [IQR] | 634 [538, 724] | 618 [520, 730] |
|  | Number of follow-up events per patient | Mean (SD) | 900.8 (1852.0) | 803.1 (1124.5) |
|  |  | Median [IQR] | 464 [299, 834] | 472 [276, 842] |
|  | Post-GLP1RA Weight Measurements | Mean (SD) | 11.96 (9.74) | 11.49 (8.82) |
|  |  | Median [IQR] | 9 [6, 15] | 8 [6, 14] |
|  | Post-treatment monthly documents per patient | Mean (SD) | 5.0 (7.8) | 4.4 (6.1) |
|  |  | Median [IQR] | 3 [2, 5] | 3 [1, 5] |
| Minimal Response  (N = 540) | Duration of prescriptions per patient in months | Mean (SD) | 12.34 (6.28) | 13.70 (6.99) |
|  |  | Median [IQR] | 12.65 [7.76, 16.71] | 13.80 [8.97, 18.49] |
|  | Number of prescriptions per patient | Mean (SD) | 9.14 (6.51) | 11.25 (9.18) |
|  |  | Median [IQR] | 8 [4, 11] | 8 [5, 15] |
|  | Follow-up duration in days, Mean (SD) | Mean (SD) | 573.75 (139.89) | 571.72 (139.85) |
|  |  | Median [IQR] | 554 [456, 681] | 553 [463, 665] |
|  | Number of follow-up events per patient | Mean (SD) | 666.3 (1391.8) | 634.6 (1217.7) |
|  |  | Median [IQR] | 367 [232, 654] | 354 [225, 618] |
|  | Post-GLP1RA Weight Measurements | Mean (SD) | 11.04 (8.17) | 9.83 (7.30) |
|  |  | Median [IQR] | 8 [6, 13] | 8 [5, 12] |
|  | Post-treatment monthly documents per patient | Mean (SD) | 5.2 (8.2) | 5.2 (8.8) |
|  |  | Median [IQR] | 3 [2, 6] | 3 [2, 6] |

**Table S8. Comparison of adverse event prevalence in the high- and moderate-response group-specific propensity-matched cohorts of tirzepatide-treated versus semaglutide-treated cohorts.** For these cohorts, 1:1 propensity matching was performed within each response group, yielding 2,318 patients in the high-response group comparison and 2,692 patients for the moderate-response group comparison. The brand name formulations for semaglutide included Ozempic, Wegovy, and Rybelsus. The brand name formulations for tirzepatide included Mounjaro and Zepbound. A -log(p-value) greater than 1.3 corresponds to a p-value less than 0.05.

| **Weight Loss Category** | **Adverse Event** | **Tirzepatide** | | **Semaglutide** | | **-log(p-value)** |
| --- | --- | --- | --- | --- | --- | --- |
|  |  | **Number of Patients** | **Prevalence**  **(% of Patients)** | **Number of Patients** | **Prevalence**  **(% of Patients)** |  |
| High Response  (N = 2318) | Nausea | 631 | 27.22 | 728 | 31.41 | 2.7094 |
|  | Fatigue | 605 | 26.1 | 689 | 29.72 | 2.1820 |
|  | Headache | 545 | 23.51 | 659 | 28.43 | 3.8133 |
|  | Constipation | 392 | 16.91 | 468 | 20.19 | 2.3372 |
|  | Diarrhea | 391 | 16.87 | 446 | 19.24 | 1.4065 |
|  | Vomiting | 297 | 12.81 | 397 | 17.13 | 4.3378 |
|  | Abdominal Pain | 335 | 14.45 | 383 | 16.52 | 1.2488 |
|  | Dizziness | 344 | 14.84 | 369 | 15.92 | 0.4834 |
|  | Heartburn | 98 | 4.23 | 94 | 4.06 | 0.0836 |
|  | Hypoglycemia | 85 | 3.67 | 85 | 3.67 | 0.0000 |
|  | Dyspepsia | 16 | 0.69 | 37 | 1.6 | 2.2421 |
|  | Decreased Appetite | 18 | 0.78 | 22 | 0.95 | 0.1981 |
|  | Flatulence | 17 | 0.73 | 20 | 0.86 | 0.1300 |
|  | Belching | 11 | 0.47 | 5 | 0.22 | 0.6767 |
|  | Abdominal Distension | 5 | 0.22 | 5 | 0.22 | 0.0000 |
|  | Gastroenteritis | 5 | 0.22 | 5 | 0.22 | 0.0000 |
|  | | | | | | |
| Moderate Response  (N = 2962) | Nausea | 486 | 16.41 | 574 | 19.38 | 2.4965 |
|  | Fatigue | 592 | 19.99 | 593 | 20.02 | 0.0000 |
|  | Headache | 491 | 16.58 | 548 | 18.5 | 1.2539 |
|  | Constipation | 355 | 11.99 | 345 | 11.65 | 0.1444 |
|  | Diarrhea | 368 | 12.42 | 369 | 12.46 | 0.0000 |
|  | Vomiting | 232 | 7.83 | 283 | 9.55 | 1.6752 |
|  | Abdominal Pain | 265 | 8.95 | 302 | 10.2 | 0.9513 |
|  | Dizziness | 264 | 8.91 | 327 | 11.04 | 2.1433 |
|  | Heartburn | 74 | 2.5 | 74 | 2.5 | 0.0000 |
|  | Hypoglycemia | 98 | 3.31 | 106 | 3.58 | 0.2090 |
|  | Dyspepsia | 17 | 0.57 | 22 | 0.74 | 0.2836 |
|  | Decreased Appetite | 20 | 0.68 | 16 | 0.54 | 0.2104 |
|  | Flatulence | 17 | 0.57 | 14 | 0.47 | 0.1434 |
|  | Belching | 13 | 0.44 | 14 | 0.47 | 0.0000 |
|  | Abdominal Distension | 5 | 0.17 | 5 | 0.17 | 0.0000 |
|  | Gastroenteritis | 5 | 0.17 | 5 | 0.17 | 0.0000 |

**Table S9. Baseline prevalence of orders or administrations for non-GLP-1RA diabetes medications or medication classes among the response-group specific propensity-matched cohorts.** For these cohorts, 1:1 propensity matching was performed within each response group, yielding different subpopulation sizes for each group: high response (N = 2318), moderate response (N = 2692), weight regain (N = 285), late response (N = 293), and minimal response (N = 540). The baseline period is defined as 5 years prior to the first GLP-1RA prescription through the date of the first GLP-1RA prescription.

| **Medication / Class** | **Weight Loss Outcome Category** | **Tirzepatide, N (%)** | **Semaglutide, N (%)** |
| --- | --- | --- | --- |
| Insulin | High Response | 372 (16.0%) | 477 (20.6%) |
|  | Moderate Response | 628 (21.2%) | 726 (24.5%) |
|  | Weight Regain | 57 (20.0%) | 65 (22.8%) |
|  | Late Response | 73 (24.9%) | 72 (24.6%) |
|  | Minimal Weight Loss | 108 (20.0%) | 138 (25.6%) |
| Metformin | High Response | 872 (37.6%) | 914 (39.4%) |
|  | Moderate Response | 1264 (42.7%) | 1458 (49.2%) |
|  | Weight Regain | 126 (44.2%) | 124 (43.5%) |
|  | Late Response | 135 (46.1%) | 136 (46.4%) |
|  | Minimal Weight Loss | 241 (44.6%) | 284 (52.6%) |
| SGLT2 Inhibitors | High Response | 208 (9.0%) | 247 (10.7%) |
|  | Moderate Response | 445 (15.0%) | 514 (17.4%) |
|  | Weight Regain | 37 (13.0%) | 33 (11.6%) |
|  | Late Response | 45 (15.4%) | 46 (15.7%) |
|  | Minimal Weight Loss | 72 (13.3%) | 97 (18.0%) |
| DPP4 Inhibitors | High Response | 93 (4.0%) | 91 (3.9%) |
|  | Moderate Response | 175 (5.9%) | 194 (6.5%) |
|  | Weight Regain | 14 (4.9%) | 20 (7.0%) |
|  | Late Response | 13 (4.4%) | 23 (7.8%) |
|  | Minimal Weight Loss | 32 (5.9%) | 43 (8.0%) |
| Sulfonylureas | High Response | 172 (7.4%) | 230 (9.9%) |
|  | Moderate Response | 322 (10.9%) | 441 (14.9%) |
|  | Weight Regain | 23 (8.1%) | 21 (7.4%) |
|  | Late Response | 35 (11.9%) | 34 (11.6%) |
|  | Minimal Weight Loss | 51 (9.4%) | 88 (16.3%) |
| Thiazolidinediones | High Response | 55 (2.4%) | 58 (2.5%) |
|  | Moderate Response | 53 (1.8%) | 78 (2.6%) |
|  | Weight Regain | <11 (<3.9%) | <11 (<3.9%) |
|  | Late Response | <11 (<3.8%) | <11 (<3.8%) |
|  | Minimal Weight Loss | 18 (3.3%) | 18 (3.3%) |
